# Pathway-specific polygenic scores for Alzheimer’s disease are associated with changes in brain structure in younger and older adults

**DOI:** 10.1093/braincomms/fcad229

**Published:** 2023-08-25

**Authors:** Judith R Harrison, Sonya F Foley, Emily Baker, Matthew Bracher-Smith, Peter Holmans, Evie Stergiakouli, David E J Linden, Xavier Caseras, Derek K Jones, Valentina Escott-Price

**Affiliations:** Institute of Neuroscience, Biomedical Research Building, Campus for Ageing and Vitality, Newcastle University, Newcastle upon Tyne, NE4 5PL, UK; Cardiff University Brain Research Imaging Centre (CUBRIC), Cardiff University, Cardiff, CF24 4HQ, UK; Cardiff University Brain Research Imaging Centre (CUBRIC), Cardiff University, Cardiff, CF24 4HQ, UK; Dementia Research Institute & MRC Centre for Neuropsychiatric Genetics and Genomics, Cardiff University, Cardiff, CF24 4HQ, UK; MRC Centre for Neuropsychiatric Genetics and Genomics, Institute of Psychological Medicine and Clinical Neurosciences, Cardiff University, Cardiff, CF24 4HQ, UK; MRC Centre for Neuropsychiatric Genetics and Genomics, Institute of Psychological Medicine and Clinical Neurosciences, Cardiff University, Cardiff, CF24 4HQ, UK; Bristol Population Health Science Institute, Bristol University, Oakfield House, Bristol, BS8 2BN, UK; MRC Integrative Epidemiology Unit, University of Bristol, Oakfield House, Bristol, BS8 2BN, UK; School for Mental Health and Neuroscience, Maastricht University, PO Box 616, 6200 MD, Maastricht, The Netherlands; MRC Centre for Neuropsychiatric Genetics and Genomics, Institute of Psychological Medicine and Clinical Neurosciences, Cardiff University, Cardiff, CF24 4HQ, UK; Cardiff University Brain Research Imaging Centre (CUBRIC), Cardiff University, Cardiff, CF24 4HQ, UK; Mary MacKillop Institute for Health Research, Australian Catholic University, 5/215 Spring St, Melbourne, VIC 3000, Australia; Dementia Research Institute & MRC Centre for Neuropsychiatric Genetics and Genomics, Cardiff University, Cardiff, CF24 4HQ, UK

**Keywords:** Alzheimer’s disease, polygenic risk scores, GWAS, *APOE*, ALSPAC

## Abstract

Genome-wide association studies have identified multiple Alzheimer’s disease risk loci with small effect sizes. Polygenic risk scores, which aggregate these variants, are associated with grey matter structural changes. However, genome-wide scores do not allow mechanistic interpretations. The present study explored associations between disease pathway-specific scores and grey matter structure in younger and older adults. Data from two separate population cohorts were used as follows: the Avon Longitudinal Study of Parents and Children, mean age 19.8, and UK Biobank, mean age 64.4 (combined *n* = 18 689). Alzheimer’s polygenic risk scores were computed using the largest genome-wide association study of clinically assessed Alzheimer’s to date. Relationships between subcortical volumes and cortical thickness, pathway-specific scores and genome-wide scores were examined. Increased pathway-specific scores were associated with reduced cortical thickness in both the younger and older cohorts. For example, the reverse cholesterol transport pathway score showed evidence of association with lower left middle temporal cortex thickness in the younger Avon participants (*P* = 0.034; beta = −0.013, CI −0.025, −0.001) and in the older UK Biobank participants (*P* = 0.019; beta = −0.003, CI −0.005, −4.56 × 10^−4^). Pathway scores were associated with smaller subcortical volumes, such as smaller hippocampal volume, in UK Biobank older adults. There was also evidence of positive association between subcortical volumes in Avon younger adults. For example, the tau protein-binding pathway score was negatively associated with left hippocampal volume in UK Biobank (*P* = 8.35 × 10^−05^; beta = −11.392, CI −17.066, −5.718) and positively associated with hippocampal volume in the Avon study (*P* = 0.040; beta = 51.952, CI 2.445, 101.460). The immune response score had a distinct pattern of association, being only associated with reduced thickness in the right posterior cingulate in older and younger adults (*P* = 0.011; beta = −0.003, CI −0.005, −0.001 in UK Biobank; *P* = 0.034; beta = −0.016, CI −0.031, −0.001 in the Avon study). The immune response score was associated with smaller subcortical volumes in the older adults, but not younger adults. The disease pathway scores showed greater evidence of association with imaging phenotypes than the genome-wide score. This suggests that pathway-specific polygenic methods may allow progress towards a mechanistic understanding of structural changes linked to polygenic risk in pre-clinical Alzheimer’s disease. Pathway-specific profiling could further define pathophysiology in individuals, moving towards precision medicine in Alzheimer’s disease.

## Teaser

This study investigated the relationship between Alzheimer’s disease pathway–specific polygenic risk scores and grey matter structure in younger and older adults. Results indicated that pathway-specific scores were associated with reduced cortical thickness and smaller subcortical volumes, particularly in the older cohort, suggesting that pathway-specific polygenic risk scores may provide more mechanistic insights.

## Graphical Abstract

**Graphical Abstract:**
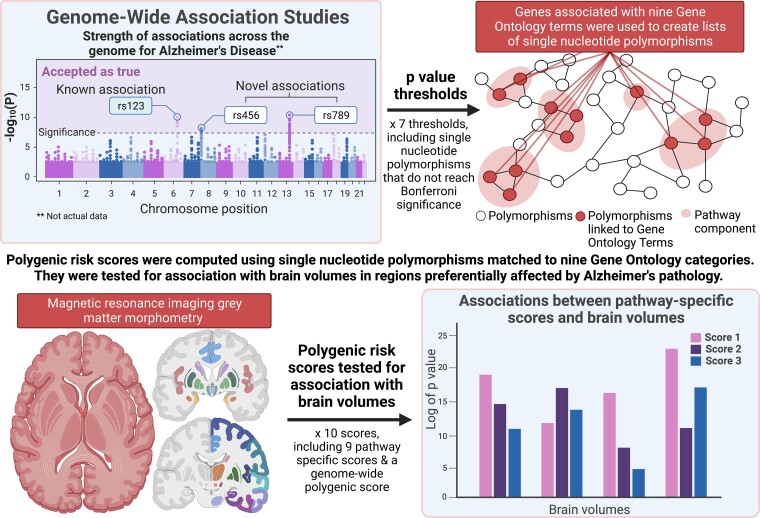

## Introduction

Alzheimer’s disease is a relentlessly progressive neurodegenerative disorder that affects between 5% and 7% of adults over 60.^1^ For a small number, early-onset Alzheimer’s is caused by mutations either in the *APP*, or Presenilin 1 and 2 genes (*PS1* and *PS2*)^2^ with an autosomal dominant mode of inheritance. For the majority, Alzheimer’s results from complex genetic and environmental interactions, with multiple genes contributing to liability. Genome-wide association studies (GWASs) have highlighted multiple Alzheimer’s risk loci of small effect in addition to the major contributor, *APOE.*^3-5^ Polygenic risk scores (PRSs) are the weighted sum of these risk loci across the genome.^6^ By quantifying the genetic burden from numerous small loci, PRS can accurately predict Alzheimer’s Disease.^7,8^ However, as PRSs aggregate risk loci, it is not possible to make mechanistic interpretations. Pathway analyses test for relationships between a phenotype and gene sets corresponding to biological pathways and have implicated areas of biology that were not previously connected to Alzheimer’s.^3,9^ Pathway-specific PRSs reflect the sum of risk loci within gene sets corresponding to biological pathways.

Structural brain imaging, particularly atrophy in medial temporal areas, is an established marker for Alzheimer’s Disease diagnosis and measurement of progression.^10^ Atrophy in the hippocampal formation and temporoparietal cortical regions are particularly likely to herald dementia symptoms.^11-15^ Subtle changes are often present years before the onset of cognitive problems.^16-18^ Pre-symptomatic carriers of rare autosomal dominant Alzheimer’s genes^19,20^ and the common Alzheimer’s risk gene *APOE*4^21-24^ show reduced hippocampal and other subcortical volumes, and cortical thinning. It is not clear how early in the life course changes appear: studies have even reported altered grey matter volumes in neonate *APOE*4 carriers.^25,26^

Combining PRS and structural neuroimaging may be particularly helpful in identifying early markers of Alzheimer’s risk,^27^ even before the amyloid deposition is detectable. A number of studies have demonstrated associations between brain structure and Alzheimer’s PRS in asymptomatic individuals.^28-33^ The findings of a small previous study suggested that pathway-specific PRS were associated with distinct patterns of neuroimaging changes.^34^ Therefore, delineating polygenic burden using disease pathway groups may enable us to detect signals associated with specific areas of biology.^3,34,35^

This study explored associations between disease pathway-specific PRS, cortical thickness and subcortical volumes in areas, such as the hippocampus, preferentially affected by Alzheimer’s pathology. We used large population samples of younger and older healthy adults, and the largest clinically-defined training dataset available.^3^ Previous studies that explored associations with pathway-specific PRS have used only 11–20 risk loci identified in earlier GWAS^36^ and had much smaller target samples.^34,37,38^ Therefore, the present study is the most powerful in the field to date.

### Hypotheses

We hypothesised that: (i) increasing genetic burden for Alzheimer’s, measured by increasing PRS, would be negatively associated with brain volumes in areas preferentially affected by Alzheimer’s pathology (hippocampal and other subcortical volumes; cortical thickness including the entorhinal cortex, the temporal pole and inferior temporal cortex); (ii) pathway-specific PRS would be associated with distinct patterns of neuroimaging changes; and (iii) changes would be evident in both younger and older healthy adults.

## Methods and materials

### Participants

Participants were recruited by the Avon Longitudinal Study of Parents and Children (ALSPAC)^39,40^ and UK Biobank.^41^ The ALSPAC sample comprised younger adults recruited to take part in population neuroimaging studies at age 19.^42,43^ Ethical approval for the study was obtained from the ALSPAC Ethics and Law Committee and the Local Research Ethics Committees. A subset of 100 000 UK Biobank participants, mean age 64, is being recalled for multimodal imaging,^41^ of which the first 20 000 datasets are analysed here. UK Biobank obtained approval from a number of external bodies.^44^ Consent for biological samples has been obtained in accordance with the Human Tissue Act (2004). This study was conducted under UK Biobank approvals for application #17044. UK Biobank participants were excluded if they self-reported a history of neurological or major psychiatric disorders at baseline or during any follow-up or had a relevant hospital admission ICD-10 code for a disorder. Excluded conditions were as follows: substance abuse/dependency, opioid dependency, alcohol dependency, bipolar disorder, schizophrenia/psychosis, neurodegenerative disorders/dementia/cognitive impairment, Parkinson’s disease, multiple sclerosis, motor neuron disease, intellectual disability or pervasive developmental disorders. Participants were excluded from ALSPAC and UK Biobank if they did not report white British and Irish descent or if they had asked to have their data removed. Data were retained if it successfully reconstructed and passed quality control.

After genotyping and imaging data quality control procedures, 517 individuals with structural T1 data remained (19.3% female, 80.7% male) in ALSPAC and 18 172 in UK Biobank (52.7% female, 47.3% male). At the time of inclusion, the average ages of ALSPAC and UK Biobank participants were 19.81 years (SD 0.02) and 64.2 (SD 7.75), respectively.

### Genotyping

ALSPAC participants were genotyped with the Illumina HumanHap550 quad genome-wide single nucleotide polymorphism (SNP) genotyping platform (Illumina Inc., San Diego, CA, USA). In UK Biobank, the first 500 participants were genotyped using the Affymetrix UK BiLEVE Axiom array and the remainder on the Affymetrix UK Biobank Axiom array. The two arrays have 95% of their content in common (https://biobank.ctsu.ox.ac.uk/crystal/ukb/docs/genotyping_sample_workflow.pdf). Quality control was completed in PLINK.^45^ Exclusions were made for as follows: (i) <97% genotyping completeness; and (ii) non-British or Irish ancestry. For both datasets, SNPs were further filtered by as follows: (i) minor allele frequency (MAF) <1%; (ii) SNP call rate <98%; and (iii) χ^2^ test for Hardy–Weinberg equilibrium *P* < 1 × 10^−4^. Imputation was performed using the prephasing/imputation approach in IMPUTE2/SHAPEIT^46,47^ with 1000 Genomes (December 2013, release 1000 Genomes haplotypes Phase I integrated variant set)^48^ as the reference dataset.

### Polygenic risk score (PRS) calculations

PRS computation was performed according to the International Schizophrenia Consortium procedure.^49^ Briefly, the discovery sample, used to select relevant SNPs, was the GWAS of Alzheimer’s Disease cases and controls conducted by Kunkle *et al*.^3^ Although larger GWAS of Alzheimer’s is available,^50^ Kunkle is the largest GWAS that used clinically-diagnosed cases (rather than self-reported family history as a proxy), and which does not include our target sample. SNPs with a low minor allele frequency (<0.01) were excluded. The data were pruned for linkage disequilibrium (LD) using the clumping function (–clump) in PLINK^45^ [parameters were r^2^ > 0.2 (–clump-r2) and 500 kilobase (–clump-kb)]. PRSs were calculated using the PLINK –score command.^45^ A previous study^28^ found that an Alzheimer’s PRS computed with *P*-value threshold (*P*^T^) of 0.001 explained the most variance in structural neuroimaging phenotypes. Therefore, the primary analysis used *P*^T^ 0.001 to select relevant SNPs from the discovery sample. Seven progressive thresholds were applied for the secondary analysis (*P* = 0.5, 0.3, 0.1, 0.01, 1 × 10^−4^, 0.1 × 10^−5^, 0.1 × 10^−6^).

In order to calculate pathway-specific PRS, relevant disease pathways were taken from the paper by Kunkle *et al*.,^3^ who detected nine Gene Ontology terms that were significantly enriched for common variants using MAGMA (Multi-marker Analysis of GenoMic Annotation).^51^ These terms are as follows: protein–lipid complex assembly; regulation of Aβ formation; protein–lipid complex; regulation of amyloid precursor protein catabolic process; tau protein binding; reverse cholesterol transport; protein–lipid complex subunit organization; plasma lipoprotein particle assembly; and activation of immune response. The genes associated with each term were used to create lists of SNPs that were matched to the discovery sample. Pathway PRSs were clumped and scored as described above. A summary of the pathways and the SNPs included in each can be found in Table 1.

**Table 1:** Significant pathways (*q*-value ≤ 0.05) from the Kunkle *et al.* MAGMA pathway analysis, *n* genes and *n* SNPs in UK Biobank and ALSPAC

Gene set no.	Pathway	Pathway description	*n* genes in the pathway in the dataset	*n* SNPs UK Biobank^a^	*n* SNPs ALSPAC^a^	Pathway includes APOE (Y/N)
1	GO:65005	Protein–lipid complex assembly	20	2215	2871	Y
2	GO:1902003	Regulation of Aβ formation	10	1700	1730	Y
3	GO:32994	Protein–lipid complex	39	4322	5663	Y
4	GO:1902991	Regulation of amyloid precursor protein catabolic process	12	1755	1913	Y
5	GO:48156	Tau protein binding	10	1709	2408	Y
6	GO:43691	Reverse cholesterol transport	17	3659	4800	Y
7	GO:71825	Protein–lipid complex subunit organization	35	1434	2301	Y
8	GO:34377	Plasma lipoprotein particle assembly	18	1556	1841	Y
9	GO:2253	Activation of immune response	382	111 843	57 493	N

Table adapted from Kunkle, B.W., Grenier-Boley, B., Sims, R. *et al*. Genetic meta-analysis of diagnosed Alzheimer’s disease identifies new risk loci and implicates Aβ, tau, immunity and lipid processing. *Nat Genet* 51, 414–430 (2019).

MAGMA, Multi-marker Analysis of GenoMic Annotation; GO, Gene Ontology.

a *n* SNPs in the UK Biobank and ALSPAC datasets prior to clumping.

### MRI data acquisition

For ALSPAC, data were acquired on a 3T General Electric HDx (GE Medical Systems) at Cardiff University Brain Research Imaging Centre (CUBRIC), Wales, UK, with an eight-channel head coil. T_1_-weighted structural images were acquired using the following parameters: 3D fast spoiled gradient echo (FSPGR) using 168–182 oblique-axial anterior commissure-posterior commissure (AC-PC) slices; 1 mm isotropic resolution; flip angle = 20°; repetition time/echo time/inverse time = 7.9 ms or 7.8 ms/3.0 ms/450 ms; slice thickness 1 mm; field of view 256 × 192 mm matrix; acquisition time = ∼6–10 minutes.^42^ For UK Biobank, data were acquired using three identical Siemens Skyra 3T scanners at UK Biobank recruitment centres, with a standard Siemens 32-channel head coil. Sagittal T_1_-weighted structural images were acquired using the following parameters: 3D Magnetization Prepared—RApid Gradient Echo (MPRAGE); *R* = 2, inverse time/repetition time = 880 ms/2000 ms; voxel size 1 × 1 × 1 mm; field of view 208 × 256 × 256 mm matrix; acquisition time = ∼5 minutes.^52^

### MRI data processing

Subcortical volumes, cortical thickness in temporal and parietal regions and intracranial volume were obtained in-house using the surface-based analysis tool FreeSurfer version 5.3 (surfer.nmr.mgh.harvard.edu).^53^ FreeSurfer has been validated as an appropriate method to segment grey matter volumes in large samples.^54^ Estimates of mean cortical thickness (mm) were based on the Desikan–Killiany atlas^55^ parcellation included in FreeSurfer. Erroneous values, due to deficient tissue segmentation or parcellation, were identified either by visual inspection or as outliers (more than 2.5 SD from the mean) and were removed from the analyses.

### Statistical analysis

Statistical analyses were conducted using R Studio v1.1.383 for Mac, www.rstudio.com.^56^ Relationships between cortical and subcortical regions of interest (ROIs) and PRS were tested using multiple linear regression. Co-variates included were as follows: age; gender; ICV; UK Biobank scanning site; UK Biobank genotyping array; principal components to adjust for population structure (10 for ALSPAC and 15 for UK Biobank, as suggested by each study^39-41^). Analyses were performed on the overall genome-wide Alzheimer’s PRS and the pathway-specific PRS separately. Resulting *P*-values were corrected for multiple comparisons of phenotype and PRS using the False Discovery Rate (FDR) in R.^56^ As a secondary analysis, results were re-analysed using a PRS that excluded *APOE* SNPs (chromosome 19, 44.4 Mb to 46.5 Mb), thereby assessing whether *APOE* explained the signal. Further analysis, using only SNPs in the *APOE* region, was performed to compare how much of the variance was explained by *APOE* alone compared to the PRS. *P*-values reported (uncorrected and FDR corrected) correspond to the PRS variable in the regression model. FDR correction was applied to the 10 PRS × *n* ROIs (nine pathway PRSs, the genome wise PRS × 26 cortical or 14 subcortical ROIs). The primary analysis, reported below, used a PRS *P*^T^ of 0.001.

## Results

### Pathway-specific polygenic scores are associated with decreased cortical thickness

**Figure 1:**
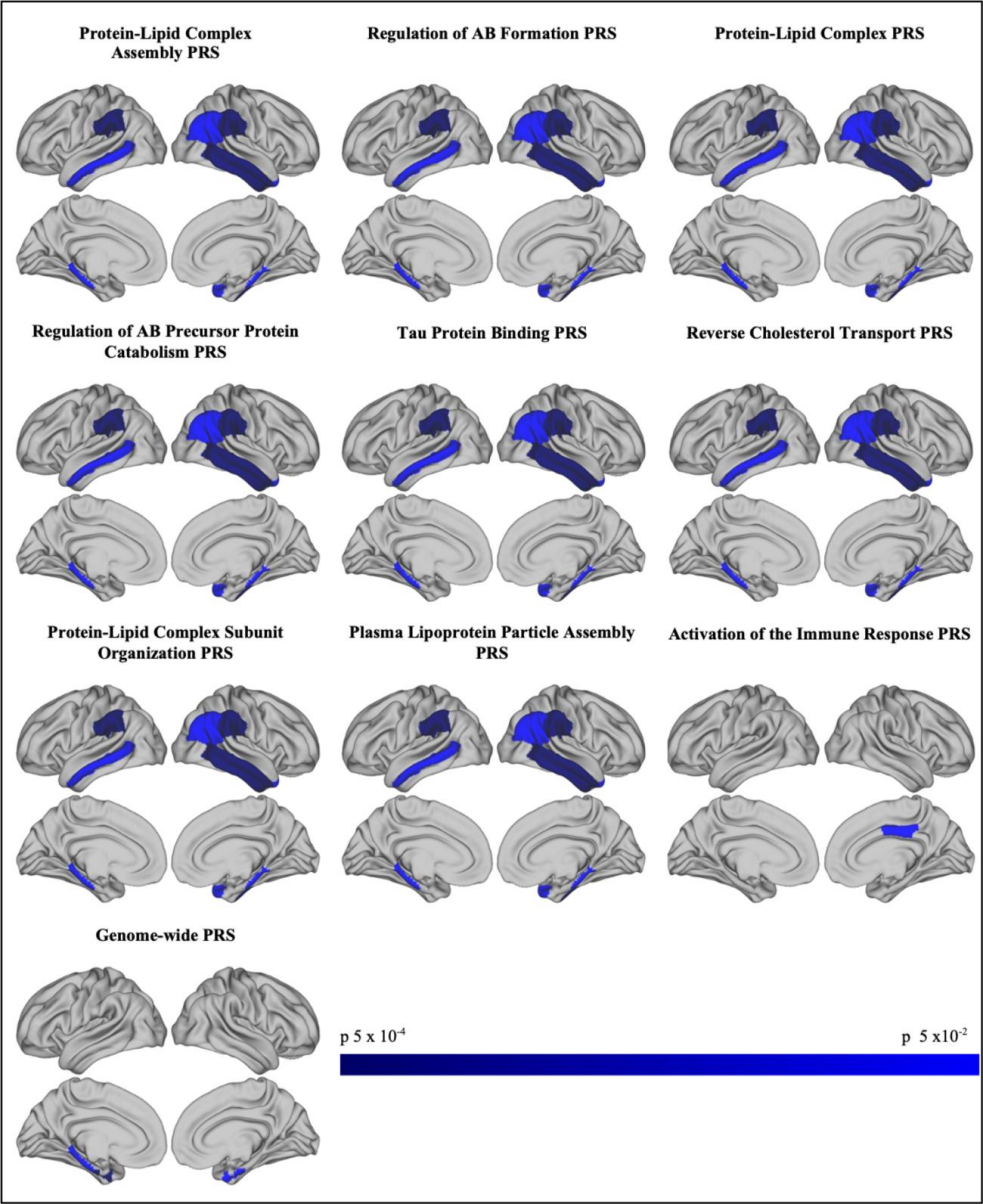
**Pathway-specific polygenic scores are associated with decreased cortical thickness in older adults (*n* = 18 172).** Relationships between cortical regions of interest (ROIs) and PRS were tested using multiple linear regression. Co-variates included: age; gender; intracranial volume; UK Biobank scanning site; UK Biobank genotyping array; principal components to adjust for population structure (15 for UK Biobank). Showing *P* < 0.05 associations between PRS (*P*^T^ = 0.001) and thickness in cortical and temporal regions of cortex in UK Biobank. Nominally significant negative correlations shown in blue. There were no positive correlations. Images created using Connectome Workbench, 56 https://www.humanconnectome.org/.^57^

Including and excluding *APOE*, there was evidence of negative association between all of the pathway-specific PRS and cortical thickness in the UK Biobank older adults. The pathway-specific scores showed a similar pattern of association. For example, the protein–lipid complex pathway was associated with reduced cortical thickness in the following regions: right inferior temporal (*P* = 0.003; beta = −0.003, CI −0.006, −0.001), right middle temporal (*P* = 0.008; beta = −0.003, CI −0.005, −0.001), right and left supra-marginal (*P* = 0.013; beta = −0.003, CI −0.005, −0.001 and *P* = 0.016; beta = −0.002, CI −0.004, −4.59 × 10^−4^, respectively), right inferior parietal (*P* = 0.025; beta = −0.002, CI −0.004, −2.82 × 10^−4^) right and left parahippocampal (*P* = 0.03; beta = −0.005, CI −0.009, −4.55 × 10^−4^ and *P* = 0.040; beta = −0.005, CI −0.010, −2.42 × 10^−4^, respectively) and right temporal pole regions (*P* = 0.041; beta = −0.005, CI −0.010, −2.26 × 10^−4^). These results were unchanged when the *APOE* region was excluded from the score, and explained more variance than the *APOE* region alone, although they did not withstand correction for multiple comparisons. Please see Supplementary Tables 1 and 2 for associations with cortical areas in the right and left hemispheres, respectively. Associations that withstood correction for multiple comparisons and those that explained more variance than *APOE* are indicated. Associations with uncorrected *P* < 0.05 are shown in Fig. 1.

**Figure 2:**
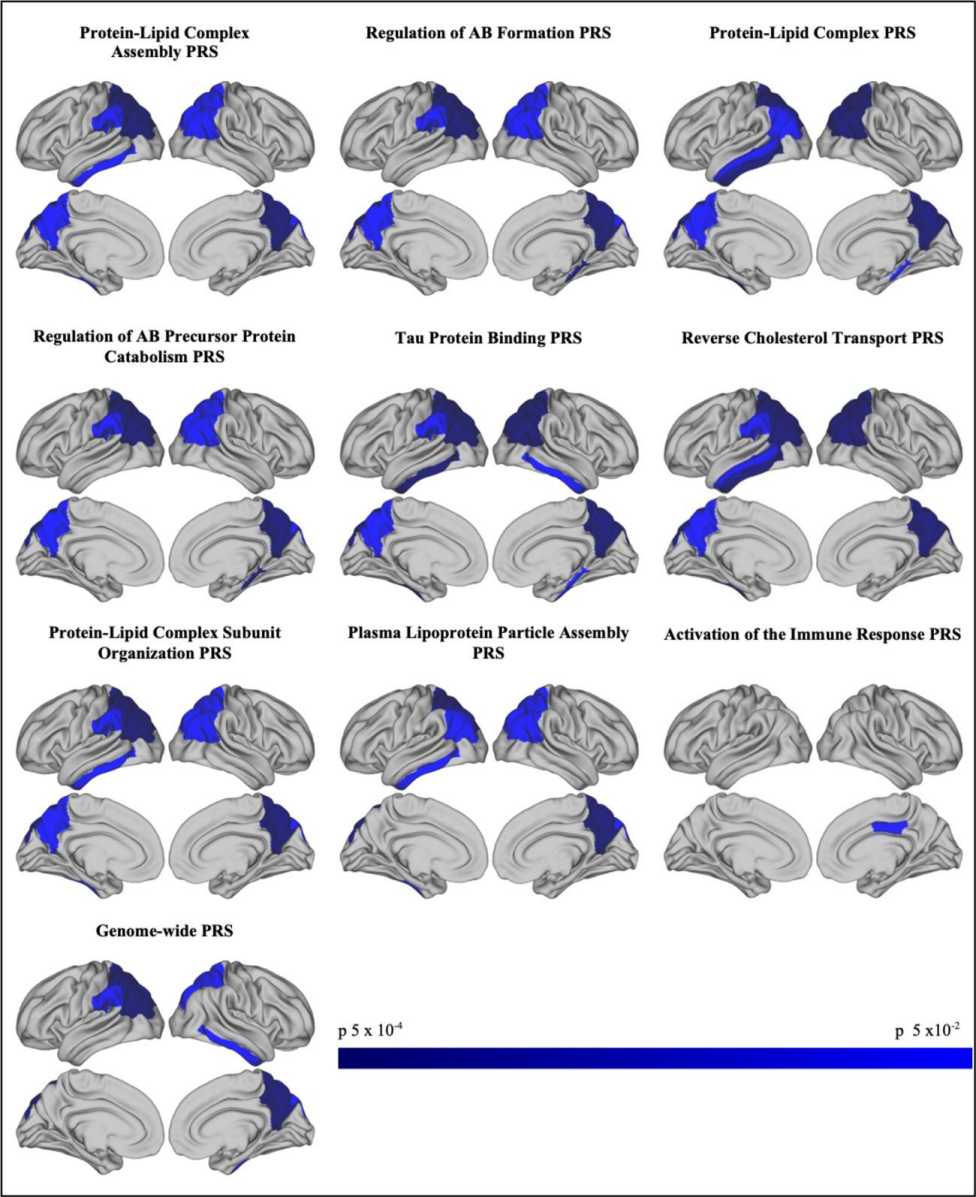
**Pathway-specific polygenic scores are associated with decreased cortical thickness in younger adults (*n* = 517).** Relationships between subcortical regions of interest (ROIs) and PRS were tested using multiple linear regression. Co-variates included: age; gender; intracranial volume; principal components to adjust for population structure (10 for ALSPAC). Showing *P* < 0.05 associations between PRS (*P*^T^ = 0.001) and thickness in cortical and temporal regions of cortex in ALSPAC. Nominally significant negative correlations shown in blue. There were no positive correlations. Images created using Connectome Workbench, 56 https://www.humanconnectome.org/.^57^

Similarly, in the ALSPAC younger adults, all pathway-specific PRSs were associated with reduced cortical thickness. Again, the pattern of association was similar between the pathway-specific PRS. For example, the reverse cholesterol transport pathway PRS was negatively associated with cortical thickness in the following areas: left inferior parietal (*P* = 0.007; beta = −0.014, CI −0.024, −0.004), left precuneus (*P* = 0.022; beta = −0.012, CI −0.022, −0.002), left superior parietal (*P* = 1.83 × 10^−4^; beta = −0.017, CI −0.026, −0.008), left supra-marginal (*P* = 0.022; beta = −0.012, CI −0.022, −0.002), left inferior temporal (*P* = 0.007; beta = −0.018, CI −0.031, −0.005), left middle temporal (*P* = 0.034; beta = −0.013, CI −0.025, −0.001), right inferior parietal (*P* = 0.008; beta = −0.015, CI −0.025, −0.004), right precuneus (*P* = 0.001; beta = −0.019, CI −0.029, −0.008) and right superior parietal (*P* = 0.003; beta = −0.014, CI −0.023, −0.005). The majority of the associations in younger adults withstood correction for multiple testing, and explained more variance than *APOE* alone. However, many attenuated when the *APOE* region was removed from the score. Associations with cortical regions in the right and left hemispheres are presented in Supplementary Tables 3 and 4. Figure 2 shows associations at uncorrected *P* < 0.05.

The pattern of association was distinctly different for the immune response PRS. This was negatively associated with the right posterior cingulate in older adults (*P* = 0.011; beta = −0.003, CI −0.005, −0.001) and ALSPAC younger adults (*P* = 0.034; beta = −0.016, CI −0.031, −0.001), and explained more variance than *APOE*. In the UK Biobank older adults, this persisted with the *APOE* region excluded, although it did not withstand FDR correction in either cohort.

The genome-wide PRS showed limited evidence of association with cortical thickness. Although the genome-wide PRS was negatively associated with cortical thickness in a number of areas, and explained more variance than *APOE* alone, when the *APOE* region was excluded from the score, only the right entorhinal cortex showed evidence of association in older adults (*P* = 0.005; beta = −0.004, CI −0.014, −0.002). After multiple comparisons correction, the genome-wide PRS showed little evidence of association with cortical thickness in either cohort.

Similarities between the results for pathways are likely due to the overlap in SNPs included, and therefore, correlations between the resulting PRS for individuals are shown in Supplementary Tables 7 and 8. Secondary analysis of cortical thickness and PRS across a range of *P*^T^ showed that the association between Alzheimer’s PRS and cortical thickness persisted, particularly with more inclusive *P*^T^ (see Supplementary Figs 1–3).

### Pathway-specific polygenic scores are associated with changes in hippocampal and other subcortical volumes

In analyses including and excluding *APOE*, the pathway-specific PRSs were negatively associated with subcortical volumes, such as the hippocampus, in the UK Biobank older adults (see Supplementary Table 5). In particular, the protein–lipid complex assembly PRS was negatively associated with volume in the left and right accumbens in older adults (*P* = 6.64 × 10^−6^; beta = −3.523, CI −5.056, −1.991 and *P* = 0.01; beta = −1.806, −3.184, −0.427, respectively), the left and right hippocampus (*P* = 8.57 × 10^−5^; beta = −11.374, CI −17.048, −5.700 and *P* = 0.01; beta = −7.522, CI −13.261, −1.783, respectively) that showed continued evidence of association after correction for multiple testing, and without the *APOE* region, and explained more variance than *APOE* alone. In the ALSPAC younger adults, there were significant positive associations between the pathway PRS and subcortical volumes. For example, the protein–lipid complex subunit organisation PRS was associated with increased volume in the left amygdala (*P* = 0.007; beta = 22.424, CI 6.278, 38.571) and the left caudate (*P* = 0.007; beta = 50.906, CI 14.113, 87.700). There was also evidence of positive associations between the several pathway PRSs, such as the reverse cholesterol transport PRS, and the left hippocampus (*P* = 0.027; beta = 55.578, CI 6.439, 104.717). Although many of the pathway PRSs explained more variance in the younger cohort than *APOE* alone, the majority of the associations in the ALSPAC younger adult cohort did not withstand correction for multiple comparisons and none were significant with *APOE* excluded (indicated on Supplementary Table 5).

The immune response PRS showed a different pattern of association. Among the UK Biobank older adults, the immune response PRS negatively associated with volume in the left hippocampus (*P* = 0.003; beta = −8.509, CI −14.183, −2.835) and right accumbens (*P* = 0.005; beta = −1.998, CI −3.376, −0.620). When the *APOE* region was excluded from the score, these results were still nominally significant. There was little evidence of association between the immune response PRS and subcortical volumes in the ALSPAC younger adults.

The genome-wide PRS was also negatively associated with the same regions and in the older adult UK Biobank group. These withstood multiple comparisons but attenuated when the *APOE* region was removed from the PRS (*P* > 0.05). In the younger adult ALSPAC cohort, there was limited evidence of association between the genome-wide PRS and any subcortical volumes (*P* > 0.05, shown on Supplementary Table 6), although direction of the effect suggested a trend towards a positive association between increased genome-wide PRS and volume in subcortical regions.

**Figure 3:**
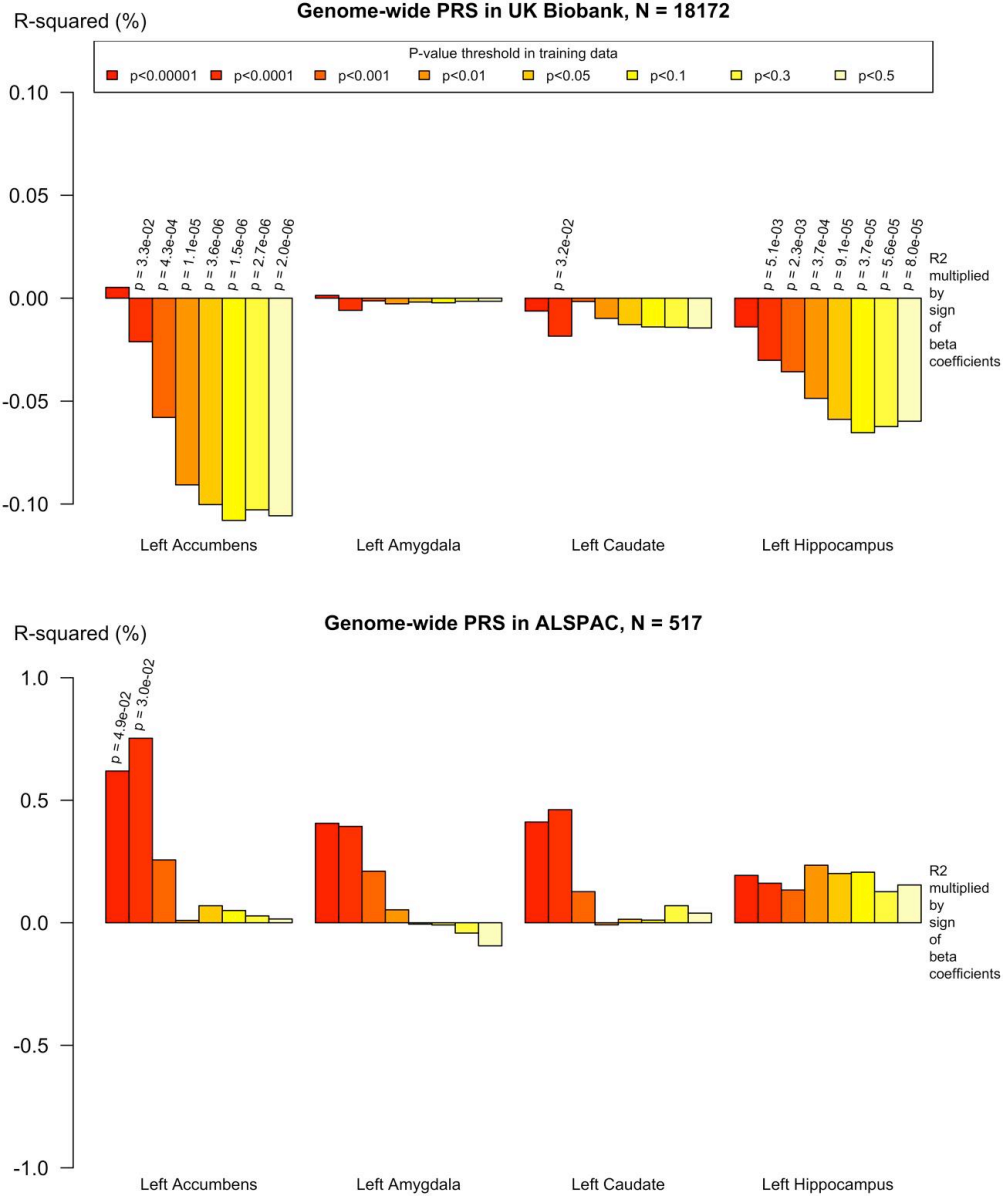
**Genome-wide PRS (including APOE) and left hippocampus, caudate, amygdala and accumbens volumes in UK Biobank and ALSPAC.** Pathway-specific polygenic scores were negatively associated with subcortical volumes in older adults (*n* = 18 172) and positively associated with subcortical volumes in younger adults (*n* = 517). Imaging phenotypes are shown on the *x*-axis, the *R*^2^ multiplied with the sign of the *B*-coefficients (positive and negative) are shown on the *y*-axis. Any nominally significant results are labelled with their nominal *P*-value. Each bar represents a version of the PRS, colour-coded by the *P*-value threshold used in the training data, shown on the legend.

**Figure 4:**
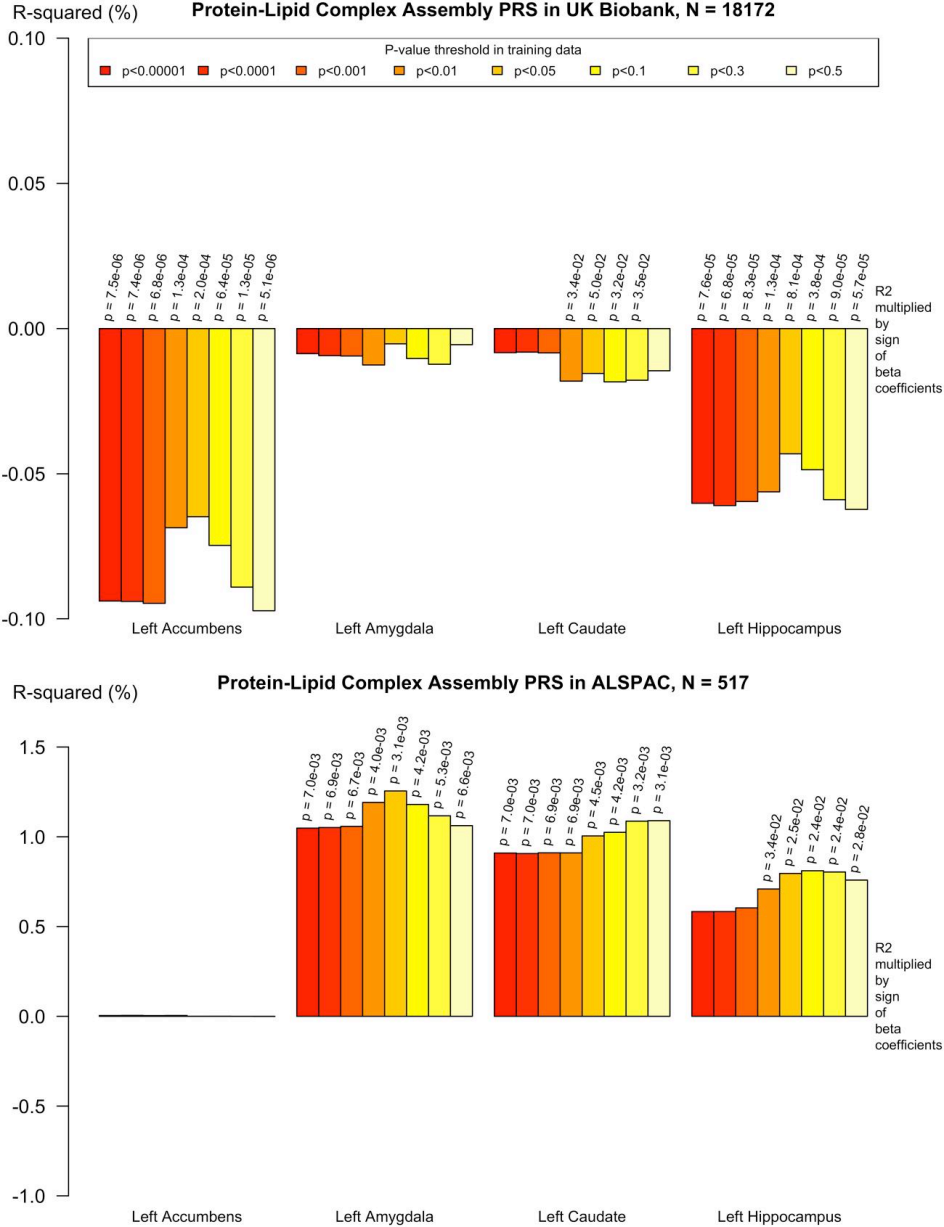
**Protein–lipid complex assembly PRS (including APOE) and left hippocampus, caudate, amygdala and accumbens volumes in UK Biobank and ALSPAC.** Pathway-specific polygenic scores were negatively associated with subcortical volumes in older adults (*n* = 18 172) and positively associated with subcortical volumes in younger adults (*n* = 517). Imaging phenotypes are shown on the *x*-axis, the *R*^2^ multiplied with the sign of the *B*-coefficients (positive and negative) are shown on the *y*-axis. Any nominally significant results are labelled with their nominal *P*-value. Each bar represents a version of the PRS, colour-coded by the *P*-value threshold used in the training data, shown on the legend.

**Figure 5:**
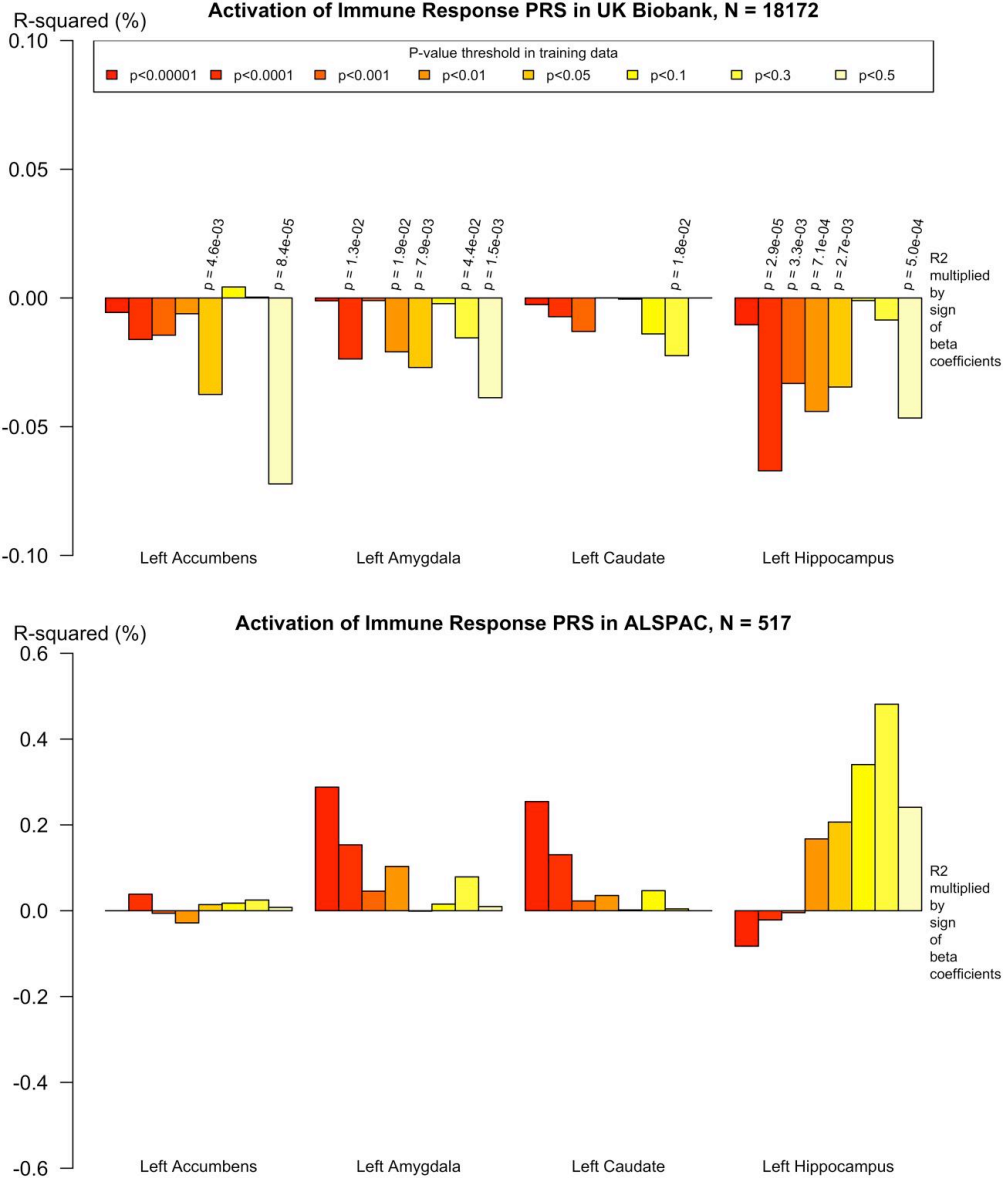
Activation of immune response PRS (including APOE) and left hippocampus, caudate, amygdala and accumbens volumes in UK Biobank and ALSPAC. Pathway-specific polygenic scores were negatively associated with subcortical volumes in older adults (*n* = 18 172) and positively associated with subcortical volumes in younger adults (*n* = 517). Imaging phenotypes are shown on the *x*-axis, the *R*^2^ multiplied with the sign of the *B*-coefficients (positive and negative) are shown on the *y*-axis. Any nominally significant results are labelled with their nominal *P*-value. Each bar represents a version of the PRS, colour-coded by the *P*-value threshold used in the training data, shown on the legend.

The results are shown in Supplementary Tables 5 and 6. Secondary analysis of subcortical volumes and PRS across a range of *P*^T^ showed that the association between Alzheimer’s PRS and decreased subcortical volumes remained, particularly with more inclusive *P*^T^. Examples of this polygenic profile are shown in Figs 3, 4 and 5.

## Discussion

Genome-wide PRSs, which aggregate Alzheimer’s risk variants, do not allow mechanistic interpretations. In this study, we found that pathway-specific PRSs were associated with lower cortical thickness and lower subcortical volumes in regions such as the hippocampus, in cognitively healthy older adults. In younger adults, increased pathway PRSs were associated with lower cortical thickness but with greater hippocampal and other subcortical volumes. The strongest evidence of association was found in left hemisphere subcortical areas and the left hippocampus, with many associations in the older adult cohort showing evidence of association beyond *APOE*.

Cortical thinning, particularly in medial temporal regions, may be an early morphometric Alzheimer’s biomarker. Lower cortical thickness has even been demonstrated in children and adolescent *APOE* carriers.^58^ This is thought to correspond to laminar thinning observed in these areas early in the disease.^59^ In contrast, normal aging has little effect on cortical thickness in medial temporal regions.^59,60^ We found negative associations between the pathway PRS and several areas in the temporal and parietal cortex. Many of these regions show the most marked cortical thinning in incipient Alzheimer’s compared to healthy older adults.^61^ Our results are in keeping with a previous study that reported that an Alzheimer’s PRS was associated with cortical thinning in these regions.^32^ In the older adults, the associations between cortical regions and pathway-specific PRS were maintained when *APOE* was excluded from the score, whereas in the younger adult cohort, the regression coefficients and *P*-values attenuated. This suggests that beyond *APOE*, polygenic burden for Alzheimer’s may not manifest in brain structure changes until later in life.

The immune response PRS showed a distinctly different pattern of association compared to the other disease pathway groups. For example, it was only associated with cortical thinning in the right posterior cingulate gyrus in older and younger adults. Both Alzheimer’s genetic risk and established Alzheimer’s have been associated with reduced thickness in the posterior cingulate.^62,63^ This was in keeping with our hypothesis and with the findings of previous studies.^34^ There was close correspondence between the genome-wide PRS and the other pathway-specific PRS in terms of the regions implicated, however, the pathway PRS often explained more variance than the genome-wide PRS. This is probably explained by the overlap in the SNPs included in each pathway, and by the *APOE* gene that is included in the full genome PRS and in the majority of the pathways (all apart from the immune response pathway). It also suggests that refining the PRS using variants implicated in pathway groups may decrease the noise inherent in the PRS approach.

Lower hippocampal volume in Alzheimer’s Disease is a robust finding.^64,65^ Whilst studies of older adults commonly report reduced hippocampal volume among *APOE* carriers, findings in younger participants are varied. Some studies report an association between *APOE* and decreased hippocampal volume in samples with mean ages ranging from 23.9 to 39.7 years,^28,66,67^ whereas others observe no significant differences in samples aged 14.4 to 28.8 years.^68-70^ Increased hippocampal volume, as we observed in the younger adult sample, has also been reported in studies of child *APOE4* carriers.^71^ Greater frontal grey matter volumes have been observed in infant *APOE4* carriers,^25^ and increased temporal grey matter volumes have been reported in children with autosomal dominant Alzheimer’s genes.^72^ Younger *APOE4* carriers also show increased activation in the hippocampus on fMRI.^73^

In the older adults, we also found an association between increased PRS and decreased volume in the nucleus accumbens, an area also implicated in Alzheimer’s. In a study of striatal morphology in Alzheimer’s cases compared to controls, Alzheimer’s patients showed significant reductions in nucleus accumbens volumes bilaterally.^74^ Other studies have identified sub-regional structural changes in the nucleus accumbens and the hippocampus in MCI and Alzheimer’s that correlated with cognitive impairment.^75^ There is also evidence of functional changes in the accumbens. For example, Kazemifar *et al*.^76^ found that activity shown on resting state MRI was significantly lower in the accumbens of Alzheimer’s cases compared to healthy controls. A study using an *APP/PS1* mouse model identified significant intracellular Aβ accumulation, increased excitability and synaptic alterations in the nucleus accumbens of transgenic mice compared to wild type.^77^

We found a preponderance of associations in subcortical regions in the left hemisphere. This is broadly consistent with the findings of previous studies. Although one group reported reduced hippocampal volume in *APOE4* compared to *APOE3* carriers on the right only,^78^ the literature predominantly observes greater evidence of changes in the left hemisphere,^28^ especially in pre-clinical or early stage Alzheimer’s,^79^ which corresponds with the findings of the present study.

We reported the association of the PRS with SNPs that are associated with Alzheimer’s at 0.001 significance level according to Kunkle *et al*.^3^ summary statistics. However, our analyses suggest that inclusion of more SNPs at less stringent thresholds increases the variance explained by the pathway-specific PRS. This provides further evidence that Alzheimer’s is a polygenic disorder as opposed to an oligogenic one as suggested by Zhang *et al*.^80^

Three previous studies have used pathway-specific PRS to explore Alzheimer’s neuroimaging phenotypes. All studied dementia-free population samples of older adults. Corlier *et al*.^37^ (sample *n* = 355) found that an immune response PRS (*n* SNPs = 11) was significantly associated with a general measure of cortical thinning. Ahmad *et al.*^38^ (sample *n* = 4521) found no significant associations between seven different pathway polygenic scores (*n* SNPs = 20), hippocampal volume and whole brain volume. Caspers *et al*. (sample *n* = 544) reported that cortical thinning associated with PRS (*n* SNPs = 20) for specific biological processes. The pathway specific effects showed a more bilateral pattern and two unique pathway specific patterns were reported, involving the superior parietal and mid/anterior cingulate regions.^34^ All of these studies had much smaller samples than the present study, and used PRS that only included loci significant after Bonferroni correction, thereby excluding relevant genetic information that is below the stringent threshold for genome-wide significance. Our study, using threshold-based PRS, may have seen significant effects where previous studies have observed mixed results.

This study benefited from using results from the largest clinical Alzheimer’s GWAS performed to date as our discovery data.^3^ Consequently, estimates of SNP effects on disease risk used in this analysis are the most accurate available, with improved power compared to previous estimates. It also benefitted from large target samples from population cohorts, which resulted in greater statistical power than previous studies. We tested the hypothesis that pathway PRSs were associated with distinct patterns of changes, as reported by a previous study^34^; therefore, we selected a variety of regions of interest that might allow patterns of associations to manifest. However, this significantly increased our burden of multiple testing. Due to the inclusion criteria of ALSPAC imaging sub-studies, males were over-represented in the younger adult cohort, and a minority of participants reported psychosis-like experiences. However, sex was included as a co-variate in the analysis, and the number of participants reporting experiences that might have met criteria for a disorder was very small.

Whilst the same PRS protocol was followed in both datasets, some heterogeneity in the genetic scores may exist and could propagate differences in the pattern of association in different datasets. It is likely that different SNPs would be excluded during quality control in each dataset, resulting in slight differences in the list of SNPs subsequently used for LD clumping. It is likely that the resulting PRS captures the variability of the important SNPs via LD, i.e. if one SNP is not available, then a SNP in LD with the missing one will be selected instead. However, this substitution may reduce the power of the analysis.

Although we divided the PRS signal into disease pathway groups, as PRS inherently pools risk variants, it remains difficult to draw firm conclusions regarding the exact molecular mechanisms underpinning the observed differences in brain structure. It is also difficult to interpret the biological or functional significance of changes seen in the younger cohort. Neurofibrillary tangles have been found in subcortical regions in younger adults,^81^ and according to the Braak staging model, areas of cortex such as the entorhinal region can be affected even earlier by this process.^82^ Amyloid deposition has been reported in younger carriers of autosomal dominant Alzheimer’s genes^83^ and in trisomy 21,^81^ however, the ALSPAC sample was probably not old enough to show detectable amyloid burden. Further longitudinal studies will be necessary to determine the effects of genetic burden for Alzheimer’s across the life course. Combining advanced MRI techniques with CSF and neuroradiology biomarkers can advance our understanding of how early changes in brain structure relate to subsequent biomarker derangement.

In conclusion, we show that increased pathway-specific PRSs were associated with reduced cortical thickness in the younger and older adults. Subcortical areas such as the hippocampus were negatively associated with pathway-specific PRS in older adults and positively associated in younger adults, in keeping with the findings of some studies in children. The immune response pathway PRS was associated with a distinct pattern of association with grey matter phenotypes, and pathway PRS generally explained more variance than the genome-wide score. This suggests that pathway-specific polygenic methods may allow progress towards a mechanistic understanding of neuroimaging changes in pre-clinical Alzheimer’s. Pathway specific profiling could further define pathophysiology in individuals, moving towards precision medicine in Alzheimer’s Disease.

## Supplementary material

Supplementary material is available at *Brain Communications* online.

## Supplementary Material

fcad229_Supplementary_Data

## Data Availability

The data used in this study are publicly available from ALSPAC and UK Biobank on request. Please note that the study websites contain details of all the data that are available through a fully searchable data dictionary and variable search tool (http://www.bristol.ac.uk/alspac/researchers/our-data/, https://www.ukbiobank.ac.uk/).
