# Supplementary material for: Pathway-specific polygenic scores for Alzheimer’s disease are associated with changes in brain structure in younger and older adults
Source: Brain Commun. 2023 Aug 25;5(5):fcad229. doi: 10.1093/braincomms/fcad229 (PMC10517196; doi:10.1093/braincomms/fcad229)
Supplement: fcad229_Supplementary_Data [file fcad229_supplementary_data.zip › Supplementary_table_legends.docx]

**Supplementary materials**

Supplementary material is available at Brain Communications online:

**Supplementary Tables 1 and 2:** Showing results for UK Biobank cortical thickness parietal & temporal regions and PRS including APOE at P^T^ 0.001

**Supplementary Tables 3 and 4:** Showing results for ALSPAC cortical thickness parietal & temporal regions and PRS including APOE at P^T^ 0.001

**Supplementary Tables 5 and 6:** Showing results for UK Biobank and ALSPAC subcortical volumes and PRS including APOE at P^T^ 0.001

**Supplementary Table 7 and 8:** Pairwise correlations between each disease pathway gene set (1-9) in ALSPAC and UK Biobank. Showing correlations with confidence intervals (CI) & p values

**Supplementary Figures 1 to 3:** Pathway specific polygenic scores were negatively associated with cortical thickness in younger and older adults, and associations persisted even at more inclusive P^T^.
