# Supplementary material for: Pathway-specific polygenic scores for Alzheimer’s disease are associated with changes in brain structure in younger and older adults
Source: Brain Commun. 2023 Aug 25;5(5):fcad229. doi: 10.1093/braincomms/fcad229 (PMC10517196; doi:10.1093/braincomms/fcad229)
Supplement: fcad229_Supplementary_Data [file fcad229_supplementary_data.zip › Supplementary Figures.pdf]

**Supplementary Materials**

**Cortical Thickness by p value Threshold in UK Biobank**

**Supplementary Figure 1.**

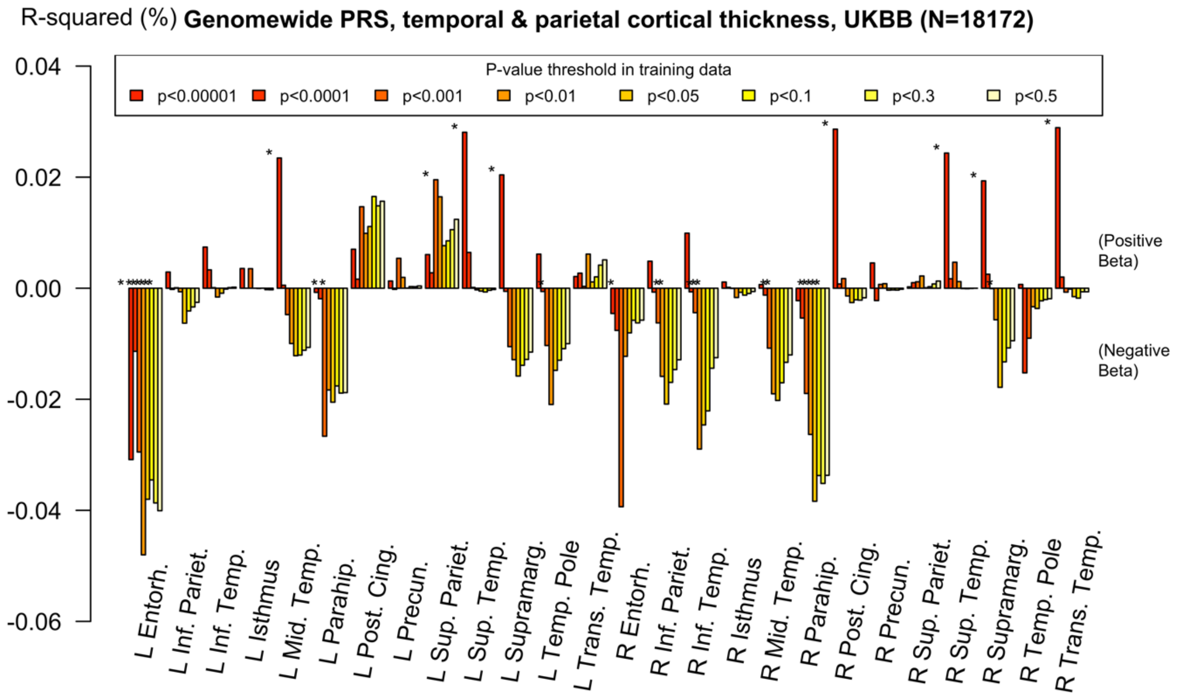


**Pathway specific polygenic scores were negatively associated with cortical thickness in young and mature adults, and associations persisted even at more inclusive $P^T$.** Figure 1 shows associations between cortical thickness regions-of-interest in the mature adult UK Biobank cohort with the Genome-wide PRS. Imaging phenotypes are shown on the X axis, the $R^2$ multiplied with the sign of the B-coefficients (positive and negative) are shown on the Y axis. Any nominally significant results are labelled with an asterisk. Each bar represents a version of the PRS and colour-coded by the p-value threshold used in the training data, shown on the legend.

**Supplementary Figure 2.**

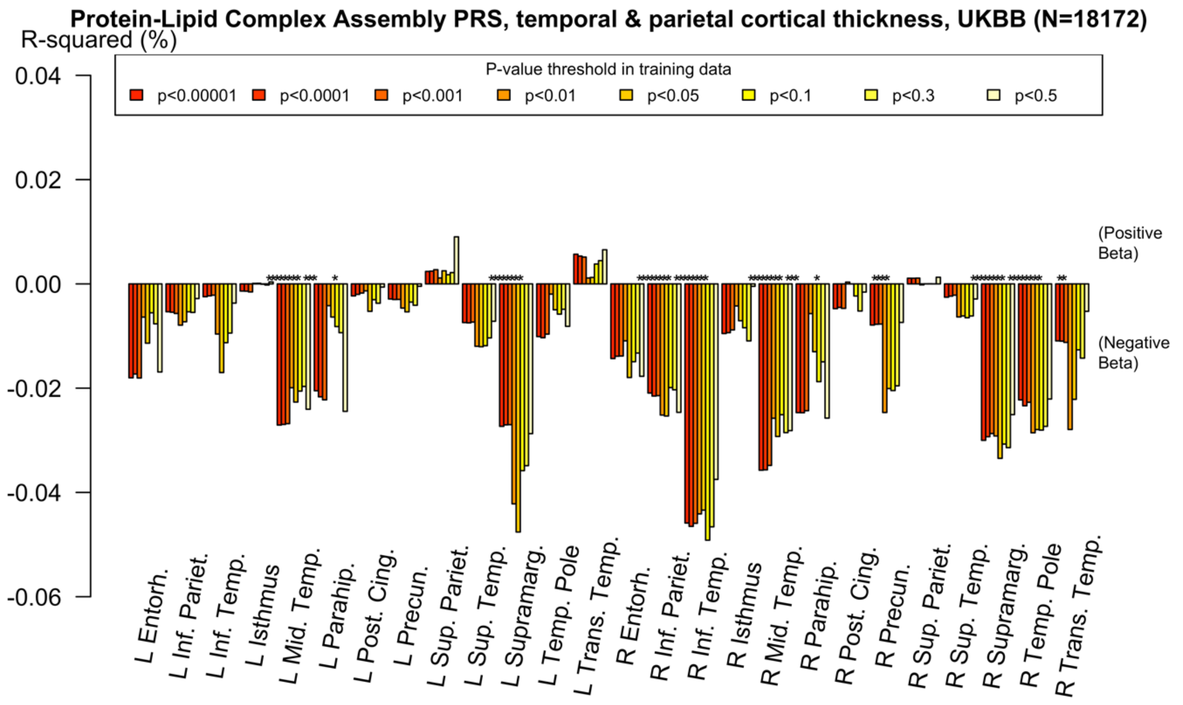


**Pathway specific polygenic scores were negatively associated with cortical thickness in young and mature adults, and associations persisted even at more inclusive $P^T$.** Figure 2 shows associations between cortical thickness regions-of-interest in the mature adult UK Biobank cohort with the Protein Lipid Complex Assembly PRS. Imaging phenotypes are shown on the X axis, the $R^2$ multiplied with the sign of the B-coefficients (positive and negative) are shown on the Y axis. Any nominally significant results are labelled with an asterisk. Each bar represents a version of the PRS and colour-coded by the p-value threshold used in the training data, shown on the legend.

**Supplementary Figure 3.**

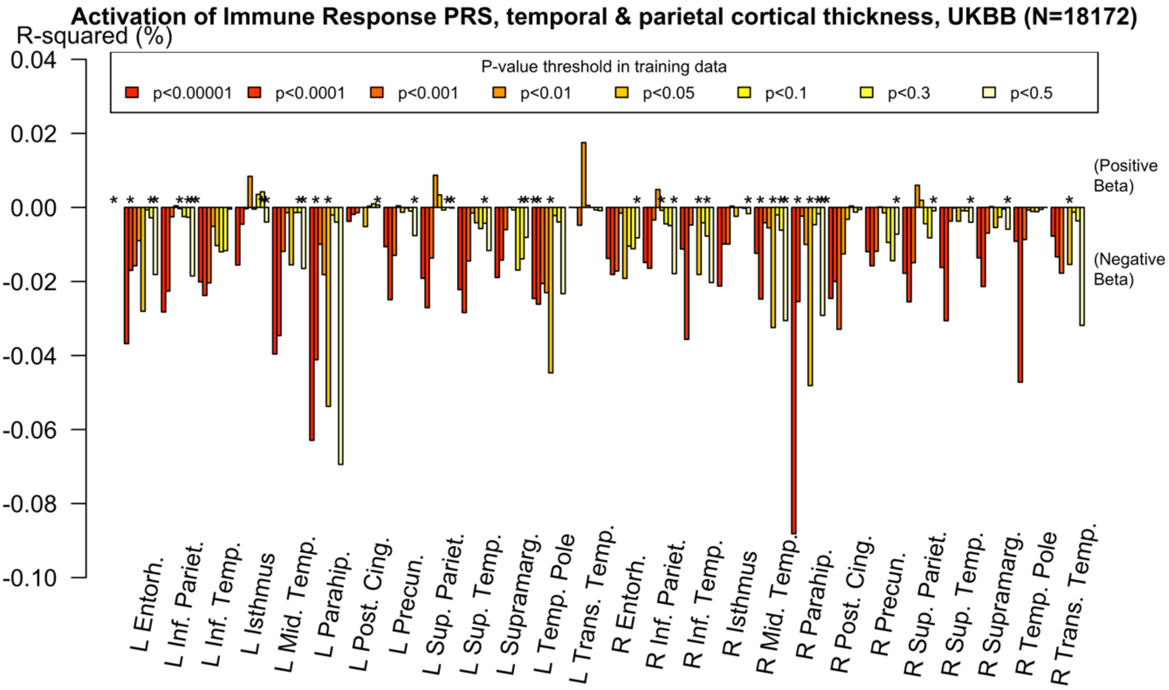


**Pathway specific polygenic scores were negatively associated with cortical thickness in young and mature adults, and associations persisted even at more inclusive $P^T$.** Figure 3 shows associations between cortical thickness regions-of-interest in the mature adult UK Biobank cohort with the Immune Response PRS. Imaging phenotypes are shown on the X axis, the $R^2$ multiplied with the sign of the B-coefficients (positive and negative) are shown on the Y axis. Any nominally significant results are labelled with an asterisk. Each bar represents a version of the PRS and colour-coded by the p-value threshold used in the training data, shown on the legend.
